# An Application of Social Vulnerability Index to Infant Mortality Rates in Ohio Using Geospatial Analysis- A Cross-Sectional Study

**DOI:** 10.1007/s10995-024-03925-3

**Published:** 2024-03-05

**Authors:** Mounika Polavarapu, Topista N. Barasa, Shipra Singh, Matthew M. Orbain, Safa Ibrahim

**Affiliations:** 1 Department of Population Health, The University of Toledo, HH 1010, Mail Stop 119 2801 W. Bancroft St, Toledo, OH 43606-3390 USA; 2 Jack Ford Urban Affairs Center, The University of Toledo, Toledo, OH 43606 USA; 3 Toledo-Lucas County Health Department, Toledo, OH 43604 USA

**Keywords:** Social Vulnerability Index, Infant Mortality rate, Relative Social Vulnerability

## Abstract

**Background:**

Ohio ranks 43rd in the nation in infant mortality rates (IMR); with IMR among non-Hispanic black infants is three times higher than white infants.

**Objective:**

To identify the social factors determining the vulnerability of Ohio counties to IMR and visualize the spatial association between relative social vulnerability and IMR at county and census tract levels.

**Methods:**

The social vulnerability index (SVI_CDC_) is a measure of the relative social vulnerability of a geographic unit. Five out of 15 social variables in the SVI_CDC_ were utilized to create a customized index for IMR (SVI_IMR_) in Ohio. The bivariate descriptive maps and spatial lag model were applied to visualize the quantitative relationship between SVI_IMR_ and IMR, accounting for the spatial autocorrelation in the data.

**Results:**

Southeastern counties in Ohio displayed highest IMRs and highest overall SVI_IMR_; specifically, highest vulnerability to poverty, no high school diploma, and mobile housing. In contrast, extreme northwestern counties exhibited high IMRs but lower overall SVI_IMR_. Spatial regression showed five clusters where vulnerability to low per capita income in one county significantly impacted IMR (*p* = 0.001) in the neighboring counties within each cluster. At the census tract-level within Lucas county, the Toledo city area (compared to the remaining county) had higher overlap between high IMR and SVI_IMR_.

**Conclusion:**

The application of SVI using geospatial techniques could identify priority areas, where social factors are increasing the vulnerability to infant mortality rates, for potential interventions that could reduce disparities through strategic and equitable policies.

## Significance

Prior studies have recognized the impact of social determinants on infant mortality and explored the spatial distribution in the state of Ohio. This study introduces a novel integration of the Social Vulnerability Index with geospatial analysis to pinpoint where social vulnerabilities overlap with high infant mortality rates. By mapping these intersections at the county and census tract levels, our research identifies specific areas in Ohio that are priority targets for intervention. This contribution not only advances the understanding of spatial patterns but also strategizes a prioritized response to addressing social determinants to reduce disparities in infant mortality.

## Background

Despite an overall 1.2% average annual decrease in infant mortality rates (IMR) from 2010 to 2019, Ohio has a higher than the national average IMR of 6.9 per 1000 live births (Ohio Department of Health, 2020). More strikingly, Black infants in Ohio are three times more likely to die compared to white infants (Ohio Department of Health, n.d.). Two-thirds of mothers who experienced infant loss predominantly lived in nine Ohio counties, one of them being Lucas County (Ohio Department of Health, n.d.). The IMR in Lucas County is continuing to rise and increased from 7.4 in 2018 to 9.4 in 2019 (Ohio Department of Health, 2020).

In 2018, IMR in the US for Black infants was 10.8 compared to white infants at 4.6 per 1000 live births (Ely & Driscoll, 2021). Although the factors contributing to observed racial disparities in IMR are complex and multifactorial, broader Social Determinants of Health (SDOH) factors are known to play a key role in shaping these disparities (Artiga et al., 2020). The SDOH are defined by the World Health Organization as the conditions in and under which people are born, grow, work, and live; and the broader set of forces and systems, including political and economic policies and systems, social policies and norms, and societal institutions that shape the conditions of daily life (World Health Organization, n.d.). Low socio-economic status has been well established as a significant risk factor at the individual level (Kim & Saada, 2013; Pabayo et al., 2019), associated with a 3% higher likelihood of infant mortality (Pabayo et al., 2019). In a recent study, household composition, specifically the family structure (single-parent family), has been linked to early age mortality among children and young adults (Braudt et al., 2019). Foreign language speaking groups, who are typically immigrants in multicultural nations such as the US, have been reported to have higher rates of infant deaths (Auger et al., 2016). Housing has also been identified as the primary social determinant of health, increasing the risk of infant mortality through housing conditions, segregation effects, and housing instability (Reece, 2021). Furthermore, not owning a car and lack of access to public transportation have contributed to the disparities in IMRs (Stevens et al., 2017). Identifying and understanding contextual social determinants at the macro level may also help explain the geographic variations in the IMR (Kim & Saada, 2013). Geographic information systems (GIS) and spatial statistical techniques have been used to present the spatiotemporal patterns of IMR and the impact of social and economic factors on its distribution (Root et al., 2020; Wang & Wu, 2020). However, such spatial relationships are yet to be studied in the context of the relative social vulnerability of geographic units.

Social Vulnerability Index, hereinafter referred to as SVI_CDC,_ was first created by the Agency for Toxic Substances and Disease Registry (ATSDR) (n.d.). The purpose of this index is to assess the relative vulnerability of geographic areas (counties, census tracts) in the US to hazardous events (e.g., natural disasters, disease outbreaks) based on social factors and to assist public health officials in identifying the communities most likely needing support before, during, and after such events (Nayak et al., 2020). The SVI_CDC_ includes fifteen social variables categorized into four themes: (i) socio-economic status, (ii) household composition and disability, (iii) minority status and language, and (iv) housing type and transportation. The application of relative social vulnerability using GIS has been expanded in recent years to study the spatiotemporal patterns of COVID-19-related cases, deaths, and vaccinations (Biggs et al., 2021; Islam et al., 2021; Neelon et al., 2021). However, this is yet to be expanded to study other health outcomes, such as mortality rates.

The primary aims of this project are to (1) identify social variables determining the relative vulnerability of counties in Ohio to IMR, (2) analyze and visualize the spatial association between the social vulnerability index and IMRs in Ohio at the county level, and (3) visualize the spatial association between social vulnerability index and IMRs at census tract level within the Lucas County.

## Methods

Cross-sectional IMR data for Ohio counties were obtained from the Environmental Public Health Tracking Network, reporting the annual average IMR between 2013 and 2017 (accessed in September 2021) (Centers for Disease Control and Prevention, n.d.). Furthermore, the IMR at the census tract level within Lucas County was obtained from Vital Statistics Records between 2017 and 2019 that were provided by the Toledo-Lucas County Health Department (n.d.). This study did not require ethical approval as it utilized publicly available aggregated data and is not based upon clinical study or patient data.

### Social Vulnerability Index by CDC (SVI_CDC_)

The SVI_CDC_ calculation includes fifteen variables organized into 4 themes. Raw data estimates and percentages for each variable are used to rank counties and census tracts (Flanagan et al., 2011). Furthermore, the counties and census tracts for the entire US and for each state are ranked against each other, ranging from 0 to 1, with higher values indicating greater vulnerability (Flanagan et al., 2011).

### Calculation of Customized Social Vulnerability Index (SVI_IMR_) for Counties within Ohio

The main effects of individual variables within each theme of SVI_CDC_ on IMR rates at the county level were assessed within Ohio, employing four multiple linear regression models. Prior to running linear regressions, the variables within each theme were tested for multicollinearity using variance inflation factor (VIF). The VIF values ranged between 1.01 and 3.91 (below the cut-off of 10), warranting no further exploration for multicollinearity.

Five out of 15 variables were significantly associated with IMR: (i) per capita income (Theme 1), (ii) percentage of persons with no high school diploma (Theme 1), (iii) percentage of persons aged 65 years and older (Theme 2), (iv) percentage of housing in structures with ten or more units (Theme 4), and (v) percentage of mobile homes (Theme 4). A customized social vulnerability index (SVI_IMR_) was calculated using the steps described in the “A Social Vulnerability Index for Disaster Management” methodology (Flanagan et al., 2011). First, a percentile rank at the county level was calculated for each of the four domains, using the sum of percentile ranks of the individual variables within those domains. The percentile ranks for Theme 3 were “0” because none of the variables within this theme were significantly associated with IMR in Ohio counties. Finally, an overall percentile rank for each county was calculated after adding the domain percentile rankings.

### Calculation of Customized Social Vulnerability Index (SVI_IMR_) for Census Tracts Within the Greater Toledo Area in Lucas County

With a similar approach described above, the five social factors were utilized to rank the census tracts within Lucas County on a scale of 0 to 1, with 1 being the highest vulnerability to infant mortality.

### Exploratory Data Analysis

Geographic boundaries of all Ohio Counties were obtained from the US Census Bureau. Exploratory data analysis was carried out to find out if there are any visual spatial patterns of IMR at the county level. Bivariate symbology in descriptive maps generated in ArcGIS Pro 2.7.1 shows the relationship between IMR and SVI_IMR_. The observations were distributed equally across the class interval, maintaining the same frequency of data per class. To determine spatial autocorrelation of IMR in Ohio counties, Local Indicators of Spatial Autocorrelation (LISA) were evaluated using the local Moran’s I statistic (Getis, 2007). The analysis was done in GeoDa 1.18.0 (Anselin, 2001; Anselin et al., 2009).

### Spatial Regression Model Estimation

The study utilized a spatial regression model that follows an autoregressive process to account for the spatial dependence among a set of observations (Anselin, 1988; Fotheringham et al., 1998). A binary connectivity matrix was constructed based on queen contiguity (Longley, 2005). The queen contiguity matrix is a first-order contiguity where two regions are defined as neighbors if they share a vertex or a common edge. An element Wij of a binary matrix (W) represents the spatial relationship of the dependent variable y between neighboring counties i and j. The connectivity matrix is constructed before the spatial regression process.

Spatial lag and error models were employed to estimate maximum likelihood (Anselin, 1988). The final selection of models was determined based on the highest log-likelihood, lowest Akaike Criterion Information, and the lowest Breusch Pagan values (Chi & Zhu et al., 2020). The spatial lag model was the best fit given the criteria. It took the form:

1 $$Y = {\rm{ }}\rho Wy + {\rm{ }}{\beta _1}{X_1} + {\rm{ }}{\beta _2}{X_2} + {\rm{ }}{\beta _3}{X_3} + {\rm{ }}{\beta _4}{X_4} + {\rm{ }}{\beta _5}{X_5} + {\rm{ }}\epsilon$$

Where $$\epsilon \sim N\left( {0,{\sigma ^2}I} \right)$$

ρ is the parameter to be estimated, W is the spatial weights matrix and Wy is the spatially lagged dependent variable. The error term ϵ is independent and identically distributed. Y was the dependent variable (IMR), and X_1 − 5_ were the independent variables (per capita income, percentage of persons with no high school diploma, percentage of persons aged 65 years and older, percentage of housing in structures with ten or more units, and percentage of mobile homes). Collectively, ρWy catered to spatial dependence.

## Results

The mean IMR was 6.72 (SD = 0.71) per 1000 live births between 2013 and 2017 in Ohio and 15.05 (SD = 11.32) per 1000 live births between 2017 and 2019 in the Greater Toledo area of Lucas County. Table 1 shows the average percentages and ranges for 14 out of 15 social factors included in the SVI_CDC_ at the county level in Ohio and the greater Toledo area in Lucas County. Additionally, mean per capita income is presented at these two geographic levels.

**Table 1:** Descriptive for infant mortality rate and social vulnerability index variables at the state and Greater Toledo area level in Ohio

	Mean	St Dev	Range		95% Confidence Interval	
			Min	Max	Lower	Upper
**Ohio**						
Infant Mortality Rate	6.72	0.71	5.49	7.94	6.57	6.87
**Theme 1: Socioeconomic Status**						
Below Poverty	14.11	4.65	4.6	30.6	13.12	15.09
Unemployed	5.49	1.62	2.2	10.5	5.15	5.83
Income	27505.07	4592.79	20,745	47,183	26531.95	28478.19
No High School Diploma	11.29	4.69	3.3	42.4	10.30	12.28
**Theme 2: Household Composition & Disability**						
Aged 65 or older	17.36	2.37	11.4	25.9	16.86	17.87
Aged 17 or younger	22.62	2.09	14.9	32.2	22.18	23.07
> Age 5 with a Disability	15.42	3.39	7.9	24.1	14.70	16.14
Single-Parent Households	8.72	1.68	3.8	12.6	8.36	9.07
**Theme 3: Minority Status & Language**						
Minority	9.98	7.70	2.1	40.8	8.35	11.61
Speaks English “Less than well”	0.54	0.45	0	2.3	0.45	0.64
**Theme 4: Housing Type & Transportation**						
Multi-Unit Structures	4.69	3.55	0.9	18.1	3.94	5.44
Mobile Homes	8.35	6.74	0.6	31.5	6.92	9.78
Crowding	1.38	0.65	0.4	4.5	1.25	1.52
No Vehicle	7.03	3.15	2.5	29.5	6.36	7.70
Group Quarters	2.95	2.86	0.5	17.2	2.34	3.55
**Greater Toledo Area**						
Infant Mortality Rate	15.05	11.32	4.07	58.82	12.48	17.62
**Theme 1: Socioeconomic Status**						
Below Poverty	23.92	17.47	0.8	78.6	20.86	26.99
Unemployed	9.90	8.12	1.1	41	8.47	11.32
Income	25398.78	11862.31	6093	77,751	23315.7	27481.86
No High School Diploma	13.40	9.15	0	40.1	11.79	15.00
**Theme 2: Household Composition & Disability**						
Aged 65 or older	14.65	5.12	0.3	34.1	13.75	15.55
Aged 17 or younger	23.52	5.88	0.6	41.5	22.48	24.55
> Age 5 with a Disability	16.77	5.96	4.9	32	15.72	17.81
Single-Parent Households	14.04	7.83	0	35.3	12.67	15.41
**Theme 3: Minority Status & Language**						
Minority	38.58	27.74	1.3	99.2	33.71	43.46
Speaks English “Less than well”	0.87	1.48	0	13.2	0.61	1.13
**Theme 4: Housing Type & Transportation**						
Multi-Unit Structures	12.00	16.62	0	90.6	9.08	14.92
Mobile Homes	1.84	4.58	0	31.2	1.04	2.64
Crowding	1.31	1.75	0	8.6	1.00	1.61
No Vehicle	13.10	12.05	0	61.6	10.99	15.22
Group Quarters	2.53	9.32	0	90.9	0.90	4.17

*Below Poverty*- Percentage of persons below poverty; *Unemployed*- Unemployment rate; *Income*- Per capita income; *No High School Diploma*- Percentage of persons with no high school diploma (age 25+); *Aged 65 or older*- Percentage of persons aged ≥ 65 years; *Aged 17 or younger*- Percentage of persons aged ≤ 17 years; *Older than Age 5 with a Disability*- Percentage of civilian noninstitutionalized population with a disability; *Single-Parent Households*- Percentage of single-parent households with children under 18 estimate; *Minority*- Percentage minority (all persons except white, non-Hispanic); *Speaks English “Less than well”*- Percentage of persons (age 5+) who speak English “less than well”; *Multi-Unit Structures*- Percentage of housing in structures with 10 or more units; *Mobile Homes*- Percentage of mobile homes; *Crowding*- Percentage of occupied housing units with more people than rooms; *No Vehicle*- Percentage of households with no vehicle available; *Group Quarters*- Percentage of persons in group quarters

The results of 4 multiple linear regressions, one for each SVI_CDC_ theme at the county level in Ohio, are presented in Table 2. In Theme 1, per capita income (*p* < 0.001) and percentage of persons with no high school diploma (*p* = 0.026) were significantly predictive of IMR. Additionally, other factors significantly predictive of IMR included the percentage of persons aged 65 and older (*p* = 0.020) within Theme 2, and the percentage of housing in structures with 10 or more units (*p* = 0.010) and the percentage of mobile homes (*p* = 0.044) within Theme 4. None of the factors within Theme 3 were significantly associated with IMR at the county level in Ohio.

**Table 2:** Results of univariate and multiple regression analyses with IMR as a dependent variable using estimated percentages of social vulnerability variables by each theme

	Coefficient	St. Error	*p*-value
**Theme 1**			
Below Poverty	0.015	0.031	0.624
Unemployed	0.115	0.074	0.121
Income	0.001	0.001	< 0.001 **
No High School Diploma	0.043	0.019	0.026*
Intercept	2.856	0.990	0.005*
**Theme 2**			
Aged 65 or older	− 0.094	0.04	0.020*
Aged 17 or Younger	− 0.028	0.04	0.505
Civilian with a Disability	0.041	0.03	0.148
Single-Parent Households	− 0.070	0.05	0.161
Intercept	9.149	1.48	< 0.001**
**Theme 3**			
Minority	0.014	0.012	0.268
Speaks English “Less than Well”	− 0.029	0.218	0.895
Intercept	6.768	0.134	< 0.001**
**Theme 4**			
Multi-Unit Structures	0.066	0.025	0.010*
Mobile Homes	0.028	0.014	0.044*
Crowding	0.086	0.153	0.577
No Vehicle	− 0.043	0.030	0.157
Group Quarters	− 0.034	0.027	0.209
Intercept	6.632	0.262	< 0.001**

(*p < 0.05, **p < 0.001)

### Bivariate Descriptive Maps- County Level in Ohio

**Fig. 1:**
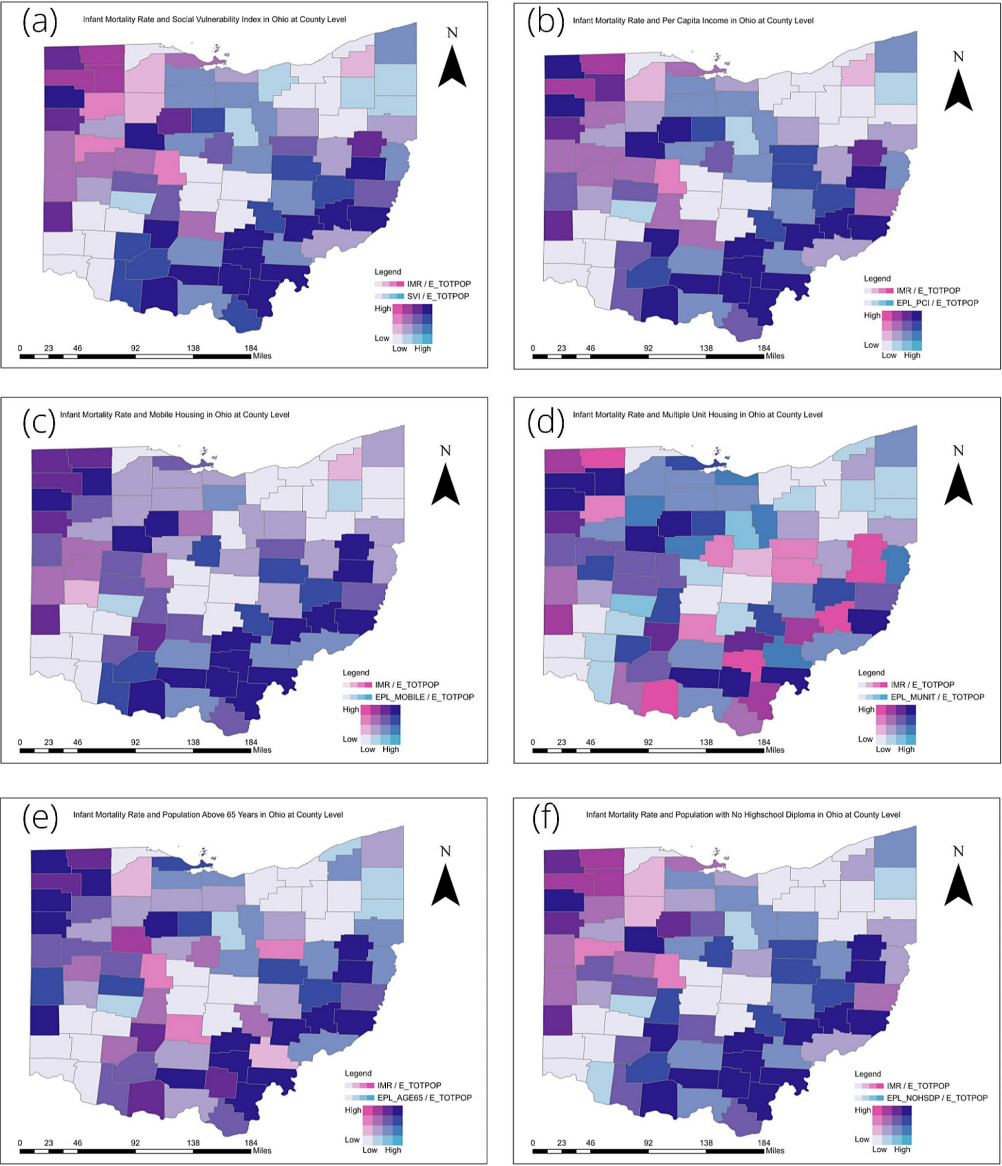
Bivariate choropleth maps (**a**) Infant Mortality Rate and Social Vulnerability Index (SVI_IMR_) in Ohio at County Level (**b**) Infant Mortality Rate and Per Capita Income in Ohio at County Level (**c**) Infant Mortality Rate and Percentage of Mobile Homes in Ohio at County Level (**d**) Infant Mortality Rate and Percentage of Housing in Structures with 10 or more Units in Ohio at County Level (**e**) Infant Mortality Rate and Percentage of households with individuals aged 65 years or older in Ohio at County Level (**f**) Infant Mortality Rate and Percentage of Persons with no High School Diploma (age 25+) in Ohio at County Level

Figure 1 shows a panel of bivariate choropleth maps. Each map within the panel simultaneously displays IMRs (Infant Mortality Rates) and the vulnerability of the region to social variables using SVI (Social Vulnerability Index) at the county-level in Ohio. The first row of colors represents high IMRs, from left to right representing low to high SVI_IMR_ (0–1, divided into four categories).

There were two notable descriptive patterns in Fig. 1. The southeast counties in Ohio (Vinton, Jackson, Meigs, Gallia) consistently displayed the highest IMRs, the highest overall SVI_IMR_, specifically the highest vulnerability to poverty, not having a high school diploma, and mobile housing. The extreme northwest counties in Ohio displayed high IMRs but lower overall SVI_IMR_, specifically lower vulnerability to poverty, not having a high school diploma, and multiunit housing.

**Fig. 2:**
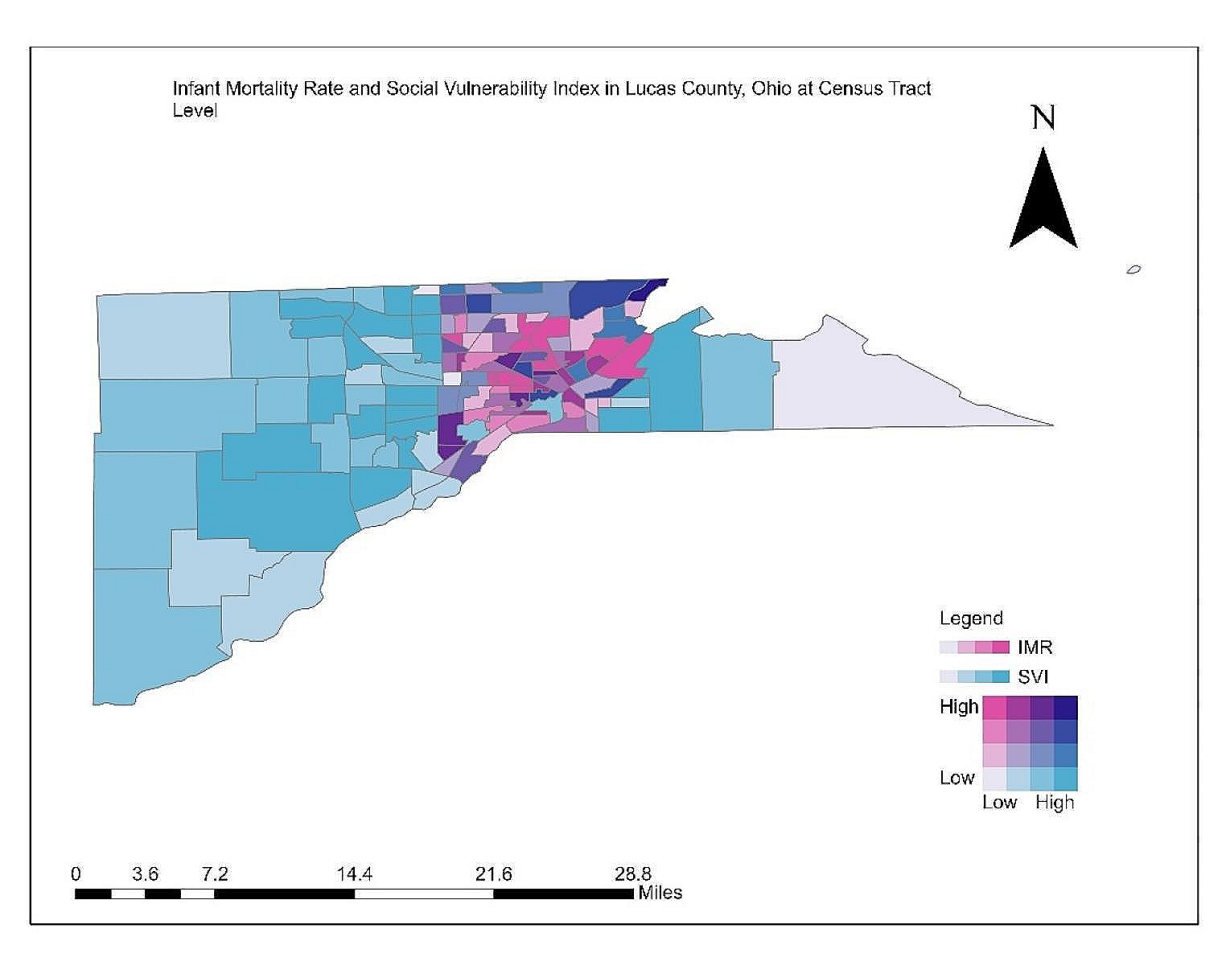
Bivariate choropleth map: infant mortality rate and social vulnerability index (SVI_IMR_) in Lucas County at census tract level

As shown in Fig. 2, most infant mortality in Lucas County occurred within Toledo city limits. Areas that are dark purple include Point Place, Glendale-Heatherdowns, and a few other smaller connecting areas. Based on these results, most areas in Lucas County that experience high levels of infant mortality do not overlap with areas of high social vulnerability, and areas that experience high levels of social vulnerability are to the East and West of the central Toledo area.

### Spatial Regression

Exploratory analysis using the local Moran’s I statistic (0.43) revealed a strong spatial autocorrelation within the residuals. There were six high-high clusters in the maps indicating the presence of spatial autocorrelation in IMR between the spatial units. Table 3 presented the results from the spatial regression model. The spatial lag model best described the relationship between IMR and the five variables at the county level in Ohio. Compared to the spatial error model, the spatial lag model had the lowest Akaike Information Criterion (-120.3), the primary guidance for model selection. In addition, the model had the lowest Breusch-Pagan value (4.9), and heteroskedasticity was insignificant (0.42). This implied there was no violation of the homoscedastic nature of the error terms. The log-likelihood (67.15) was also higher for the spatial lag model. A total of 60% of the variance in IMR at the county level in Ohio was explained by the spatial regression model. Within the model, only per capita income (*p* = 0.001) was significantly associated with IMR.

**Table 3:** Spatial regression model

Variable	Coefficient	Std. Error	*p*-value
Weighted IMR	0.609	0.101	< 0.001**
Constant	1.064	0.274	< 0.001**
Per Capita Income	− 0.240	0.075	0.001*
No High School Diploma	0.065	0.066	0.323
Multiunit Housing	− 0.003	0.051	0.950
Mobile Housing	0.121	0.064	0.057
Age above 65 years	− 0.016	0.041	0.700

(*p < 0.05, **p < 0.001)

**Fig. 3:**
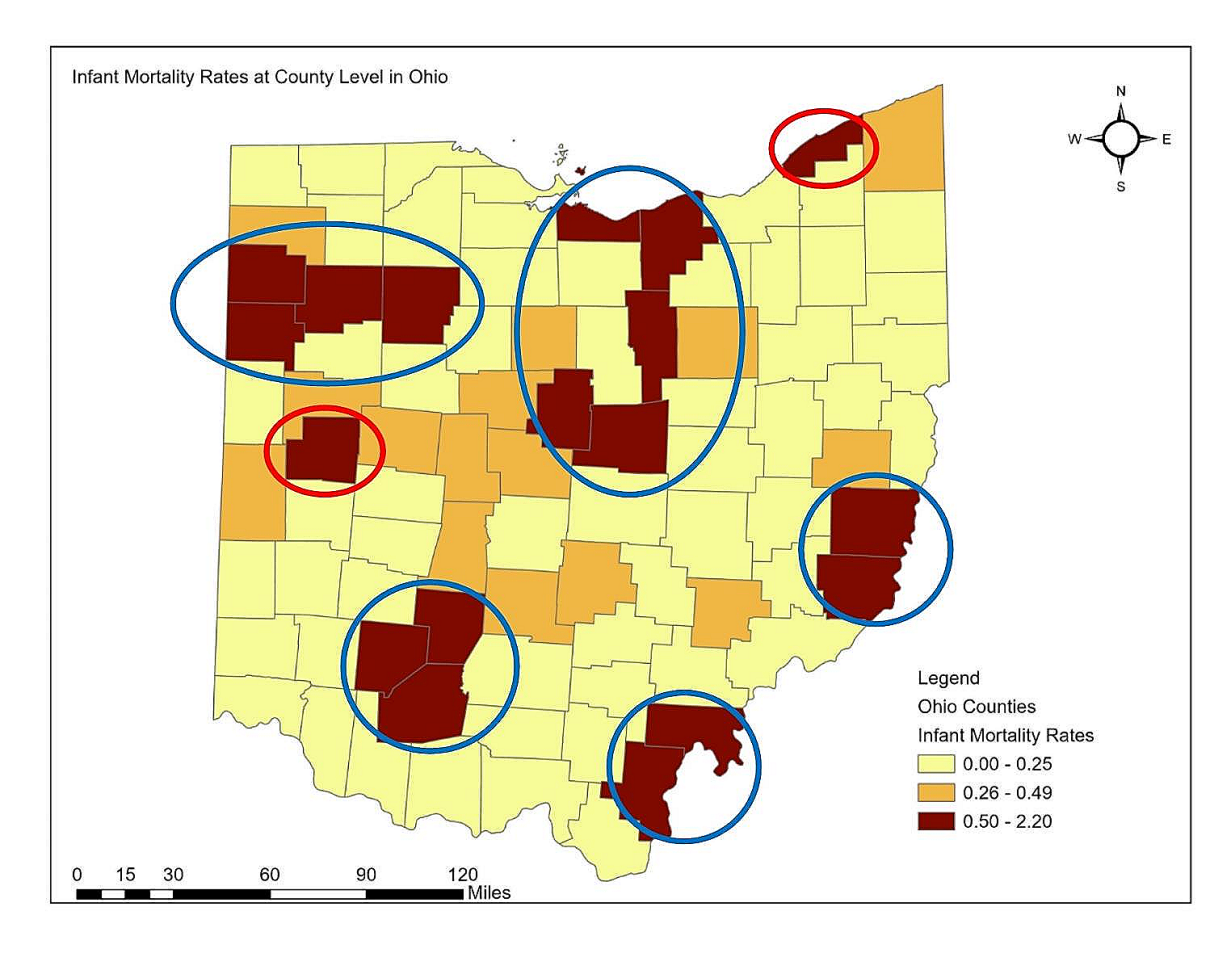
Spatial clustering of infant mortality rates

Figure 3 represents the spatial distribution of IMR in Ohio at the County level. There are seven significant clusters from the visualization (Southeast, southwest, north, northwest, and parts of central counties) where the covariates explain high IMR within the model. In 5 clusters (circled in blue in Fig. 3) among the seven, one county’s vulnerability to low per capita income significantly impacted high IMRs in the neighboring counties within each cluster. In the remaining two (circled in red in Fig. 3), the cluster consisted of one county where the per capita income significantly impacted the IMR only within that county, with low impact on neighboring counties.

## Discussion

In 2021, Ohio ranked 43rd in the nation in IMR, with staggering racial and ethnic disparities (America’s Health Rankings, 2021). The state has identified infant mortality as a priority in its most recent state health improvement plan (Ohio Department of Health, 2017). The Ohio Equity Institute (OEI), a collaboration between the Ohio Department of Health and local partners, was created in 2012 and selected nine counties in Ohio as priority counties with the greatest racial disparities to address the biggest drivers of inequities in poor birth outcomes and infant mortality (Ohio Equity Institute, 2021). Lucas County, one of the selected priority counties by the OEI, reported having IMR during 2013–2017 more than double in comparison to overall IMR in Ohio. In addition, for each social variable included in the SVI_CDC_, Lucas County reported a more detrimental exposure to the vulnerability. This highlights the critical need to understand the drivers of observed disparities in birth outcomes at a more granular level to address the need of the communities appropriately.

Ours is the first study to utilize the relative social vulnerabilities of geographic areas to identify the priority areas for public health action in reducing IMR. We developed a customized social vulnerability index (SVI_IMR_) for Ohio at the county level from the SVI_CDC_. The five variables that were included in the customized SVI_IMR_ were per capita income, percentage of persons with no high school diploma, percentage of persons aged 65 years and older, percentage of housing in structures with ten or more units, and percentage of mobile homes.

The five social factors in the SVI_IMR_ have been investigated and established as determinants of IMR in the literature. Multiple studies have reported the association between lower socio-economic status and poorer birth outcomes, including lower birth weight and higher IMR (Martinson & Reichman, 2016; Mohamoud et al., 2019; Scharber, 2014). Lower educational attainment has been associated with lower-paying employment opportunities with fewer benefits, living in underserved neighborhoods, and experiencing greater housing instability (Stevens et al., 2017).

Lower IMRs associated with a higher percentage of persons aged 65 years and older could be interpreted in the context of multigenerational homes. Multigenerational households provide a safe environment and higher quality of childcare and infant development in addition to increasing psychological, social, and financial capital to help with health improvements and IMR (Muennig et al., 2018; Sadruddin et al., 2019).

Past studies have shown that housing instability and the lack of consistent and reliable residence can impact maternal and child health (Leifheit et al., 2020; Reece, 2021). About 4% of renters in Ohio experienced an eviction every year from 2002 to 2016 (Steinman et al., 2018). Most low-income women experiencing housing instability live in the inner city with higher rates of poverty, violence, illicit drug use, and decreased transportation access, which has been linked to higher maternal and infant mortality rates (Archer et al., 2017).

Visualization of the spatial association between SVI_IMR_ and IMR in Ohio at the county level revealed two distinguished descriptive patterns. The southeastern and northwestern counties in Ohio both have high rates of IMRs. However, there is a simultaneous higher vulnerability to social factors in southeastern counties than in other counties. On the other hand, the northwestern counties displayed lower vulnerabilities to social factors. This suggests that in the efforts to reduce IMR in Ohio, southwestern counties may be important targets for increased interventions and resource allocation towards addressing poverty, no high school diploma, and mobile housing units. In contrast, in the northwestern counties that include Lucas County, efforts to address the issues of poverty, not having a high school diploma, and multiunit housing may not yield a proportional reduction in IMR.

The counties with high IMRs that do not correspondingly exhibit high levels of social vulnerability prompt a different yet critical examination of policy and program implications, necessitating innovative community-centric approaches. By mobilizing existing resources and leveraging community assets, such programs and policies could aim at enhancing access to and quality of health services independent of the broader geographic social vulnerability context (Pies et al., 2016). Specifically, the strategies could be holistic, bolstering prenatal care through local support networks and improving healthcare infrastructure (Fareed et al., 2022; Johnson et al., 2023). CDC has suggested the effectiveness of such customized approaches in boosting COVID-19 vaccination rates in similar contexts of low social vulnerability index (Barry et al., 2021).

A similar visualization at a much smaller geographic unit of census tracts within a county has displayed a contrasting pattern where the overlap of high social vulnerability (overall SVI_IMR_ closer to 1) with high IMR was observed in the urban census tracts. Conversely, at the county level analysis, such overlap was more pronounced in rural southeastern counties. This observed contrast emphasizes the need to study health outcomes in relation to social factors at a much smaller geographic level instead of a broader county level. Public health experts have advocated such an approach, where allowing smaller communities to understand their chronic disease burden and the associated social determinants has been suggested to enhance the equitable use of resources (Hacker et al., 2021). A spatiotemporal analysis of IMR in Ohio between 2008 and 2015 revealed the clustering of infant deaths in the urban regions of Ohio (Root et al., 2020). This study utilized geocoded locations of each infant death, unlike the current study, which used aggregated data for a geographic unit (Root et al., 2020).

Spatial regression analysis at the county level in Ohio revealed seven significant clusters where high IMRs were significantly explained by low per capita income. Clustering implies that low per capita income in one county significantly impacts high IMR in the neighboring counties within each cluster. This spillover effect could be explained by the similar lack of resources and opportunities being shared by neighboring counties. The possible limitations of this study relate to the availability of data at the time of analysis. The IMRs (2013–2017) and SVI (2014–2018) data correspond to the pre-COVID-19 pandemic time period. The COVID-19 pandemic has significantly impacted the social vulnerabilities of communities and IMRs (Paremoer et al., 2021; Orgera et al., 2021; Murphy et al., 2021; Shapira et al., 2021). Our study utilized cross-sectional attributes of geographical areas and did not take into consideration the temporal changes in both social factors and IMR. In the future, the changes in social vulnerabilities with time must be taken into consideration. Furthermore, spatial regression analysis could not be expanded to the census tract level in Lucas County because of the inability to perform a log transformation of IMR for census tracts that reported zero rates during that time period.

In summary, the relative social vulnerability could enable the identification of priority counties and, within counties, priority census tracts to address social factors in reducing IMR. Geospatial inquiries have historically informed policies to prevent maternal and newborn deaths through interventions such as housing vouchers, expansion of low-income housing availability, and rent support programs. Prioritizing resource allocation for the most vulnerable regions could promote equity by focusing on disadvantaged communities where interventions are likely to be most impactful. However, relying solely on the bias of geographic social vulnerability may overlook communities where IMR is high due to other complex factors beyond what social vulnerability indices alone can capture. Recent studies examining the SVI_CDC_ ranking method have shown the association between social vulnerability and the COVID-19 pandemic. Increased risk of COVID-19-related death was found in counties with SVI_CDC_ ≥ 0.6, approximately 40% of US counties (Freese et al., 2021). Likewise, studies have leveraged SVI_CDC_ to identify COVID-19 spatial clusters, which were classified as priority areas for resource allocation (Dasgupta et al., 2020; Freese et al., 2021). Hence, future research should focus on utilizing the advantages of spatial analysis to align interventions with the multifaceted needs of communities. This could ensure approaches to reducing disparities in infant mortality rates, moving away from blanket applications for strategic and equitable policies and practices.

## Data Availability

Will be provided upon request.

## Code Availability

Will be provided upon request.
